# Books and Authors

**Published:** 1910-05

**Authors:** 


### BOOKS AND AUTHORS.

**Medical Diagnosis.** By **Charles Lyman Greene,** M.D., Professor of Medicine and Chief of the Department in the College of Medicine, University of Medicine, Minneapolis. Third edition. 12 mo, pp. 725. Illustrated. Philadelphia: P. Blakiston's Son & Co. (Leather, $3.50.)

With the first appearance of this book, now some three years ago, it took favor with the medical profession. Of all the manuals on medical diagnosis that have appeared in recent years, and they are many, this perhaps is the most popular; at least, there is none that excels it in this regard. The reason or reasons are not far to seek: it is compact, yet sufficiently comprehensive to meet all the demands of a manual; it is well printed with subheads in heavy faced type, and with elaborate marginal notes and references; making possible quick examination of topics; it is handsomely and substantially bound in flexible leather with gilt edges and rounded corners; and, moreover, its contents table and index are well prepared, even elaborately made up.

This edition presents the book in thorough revision, with such additions as have become necessary to bring it forward to the immediate present. It has been remodeled and rewritten to quite an extent, some new illustrations have been introduced, and altogether is a work that commends itself to both student and practitioner of medicine.

**Progressive Medicine,** Vol. 12, March, 1910. A Quarterly Digest of Advances, Discoveries and Improvements in the Medical and Surgical Sciences. Edited by **Hobart Amory Hare,** M.D., Professor of Therapeutics and Materia Medica in the Jefferson Medical College of Philadelphia. Lea & Febiger, Philadelphia and New York. (Per annum, paper bound, $6.00; cloth, $9.00.)

This number deals with surgery of the head, neck and thorax, by Charles H. Frazier; infectious diseases, including acute rheumatism, croupous pneumonia, and influenza, by John Ruhräh; diseases of children, by Floyd M. Crandall; rhinology and laryngology, by D. Braden Kyle; and otology, by Arthur B. Duel. The book is filled with interesting material gleaned from the more recent literature by men who know how to select and to condense, thus insuring the latest and best. Hence the book becomes one of the most useful reference volumes of the present day.

**Fifth annual report of the Henry Phipps Institute for the Study, Treatment and Prevention of Tuberculosis.** February 1, 1907, to February 1, 1908. Edited by **Joseph Walsh,** A.M., M.D.

The good work of this institution done during its fifth year February 1, 1907, to February 1, 1908 is set forth in this well printed volume of 460 pages. In this period 1,475 persons were admitted for treatment; in the five years since its founding 5,849 individuals have been admitted for treatment. The enormous work relating to the recording, classifying, and treating this number of tuberculosis patients will be comprehended by every physician familiar with hospital work. Again, it will be appreciated that this institute is one of the most useful philanthropies in the whole country.

---

**The Practical Medicine Series.** Ten volumes. Under the general editorial charge of **Gustavus P. Head,** M.D., Professor of Laryngology and Rhinology in the Chicago Post-Graduate Medical School. Vol. X. Nervous and Mental Diseases. Edited by **Hugh T. Patrick,** M.D., and **Charles L. Mix,** M.D. Series 1909. Chicago. The Year Book Publishers. (Price, $1.25; entire series, $10.00.)

A careful digest of the improvements in nervous and mental diseases for the year, such as we find this book to be, affords the general practitioner a vast amount of information in shape for ready reference. The compilers are men of experience and they have brought out the salient features of the year's literature in excellent form. The book contains a goodly number of useful illustrations. We commend the volume to all interested in mental and nervous diseases.

---

**International Clinics.** A Quarterly of Illustrated Clinical Lectures and especially prepared articles on Treatment, Medicine, Surgery, Neurology, Pediatrics, Obstetrics, Gynecology, Orthopedics, Pathology, Dermatology, Ophthalmology, Otology, Rhinology, Laryngology, Hygiene, etc. Edited by **Henry W. Cattell, M.D.** Vol. I, twentieth series. Philadelphia and London: J. B. Lippincott Co. 1910. (Cloth, $2.00.)

Under the head of special articles in this volume we find the serum diagnosis of syphilis, by Homer F. Swift; further studies of the serum diagnosis of syphilis with special reference to the antihuman hemolytic system, by Hideyo Noguchi; and the newer diagnostic methods of syphilis of the nervous system, by B. Sachs. Relating to diagnosis and treatment are symptomatology of pellagra, by J. J. Watson; treatment of pellagra, by James M. King; and tuberculins and their diagnostic and therapeutic use. In medicine first comes an article giving recent additions to our knowledge of purin metabolism and their bearing on the problems of gout, by H. Gideon Wells; and then one on chronic mucous colitis, by Dudley Fulton. Two articles appear under surgery, one relating to the diagnostic value and therapeutics of bismuth paste in chronic suppuration, by Emil G. Beck; and the other deals with tuberculosis of the thyroid gland, giving the report of a case, by A. E. Halstead. Gynecology contributes a paper on the progress of gynecology and abdominal surgery during the last twenty years, by A. Lapthorn Smith; also one on the hygiene of menstruation, by Ernest Bowen Young. The single article on pediatrics relates to eye strain among school children and is written by Aaron Brav. In neurology is a paper on tabes dorsalis giving its rational treatment in the light of its real pathogenesis, by Tom A. Williams. Anatomy contributes a paper giving an investigation of the portio vaginalis of the uterus in relation to conception, by June Kichi Kinura. Under the head of miscellaneous topics is an article on the course of postgraduate study of the American Medical Association, by John H. Blackburn. The book closes with progress of medicine during the year 1909,—treatment by A. A. Stevens; medicine, by John H. Musser; and surgery, by Joseph C. Bloodgood. It is a volume filled with good things.

---

**Anatomy and Physiology for Nurses.** By **LeRoy Lewis**, M.D., Surgeon to and Lecturer on Anatomy and Physiology for Nurses at the Lewis Hospital, Bay City, Mich. Second edition. 12mo, pp. 344. Illustrated. Philadelphia and London: W. B. Saunders Co. 1910. (Cloth, $1.75.)

The thought that prompted the preparation of this book was an excellent one—namely, to afford nurses a concise work on anatomy and physiology that would give sufficient information without being too technical. The book has stood the test of two editions and six separate printings, indicating the approval of those for whom it was specially intended. This revision contains much new material distributed throughout the text and one entire new chapter,—that on the male organs of generation. Pupil nurses will find the book adapted to their requirements, and it will prove useful to graduate nurses for study and reference.

